# Supplementary material for: An Energy Modulation Interrogation Technique for Monitoring the Adhesive Joint Integrity Using the Full Spectral Response of Fiber Bragg Grating Sensors
Source: Sensors (Basel). 2024 Dec 25;25(1):36. doi: 10.3390/s25010036 (PMC11723423; doi:10.3390/s25010036)
Supplement: Supplementary file 1 [file sensors-25-00036-s001.zip › sensors-3353397-supplementary.pdf]

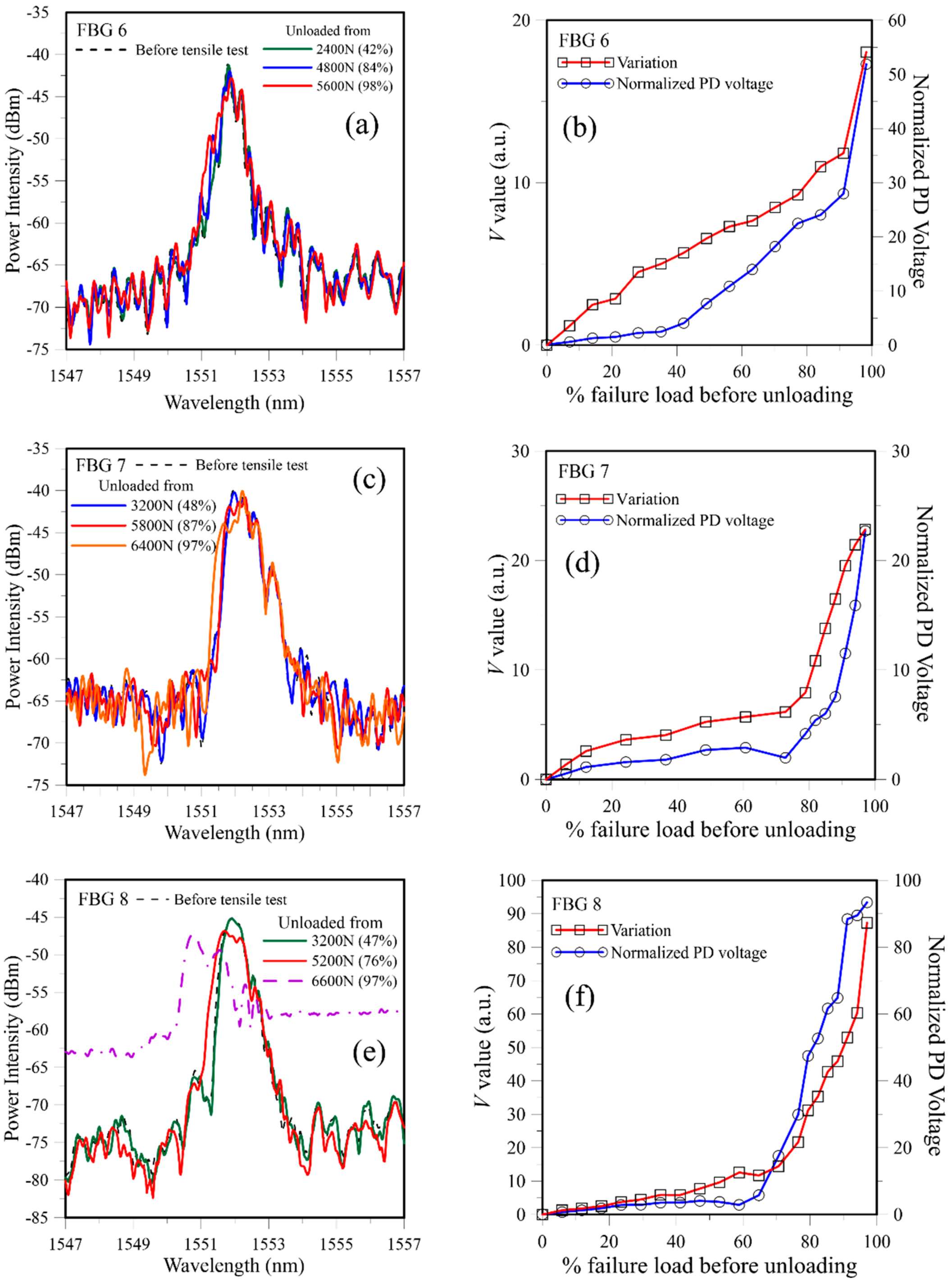

Figure S1. Evolution of the load-free spectra during tensile tests in (a) FBG 6; (c) FBG 7 and (e) FBG8; and comparison of the corresponding V values and simulated PD output for filters FWHM = 5 nm and initial overlap of 0.4 for (b) FBG6; (d) FBG 7, and (f) FBG8. (the bracketed numbers after the loading values in the figure legends indicated the loading as a percentage of the failure load).
